# Supplementary material for: Multi-party co-signature scheme based on SM2
Source: PLoS One. 2023 Feb 6;18(2):e0268245. doi: 10.1371/journal.pone.0268245 (PMC9901816; doi:10.1371/journal.pone.0268245)
Supplement: Attachment — Submitted filename: Plos-d-21-40703-Response to Reviewers.docx [file pone.0268245.s001.docx]

## Responses to reviews

We thank the editor and the reviewers for their insightful comments. We are glad that they have found the current research to be valuable, the method used reliable to provide novel results and the discussion substantial to explain the results. In sum, we thank the reviewers for noting that we have filled the research gap by asking an important question that has not yet been thoroughly addressed in the existing literature.

We have further revised the manuscript in light of the review comments. Below, we summarise the major changes we have made to the manuscript, and then we detail our responses to the review comments point by point.

We numbered the comments made by reviewers and started with bold letters " ***Q1***" for the ﬁrst reviewer, "***Q2***" for the second reviewer, "***Q3***" for the third reviewer, and "***Q4***" for the fourth reviewer. In addition, responses to the comments are started with the bold letter "**Response to ***". In order to make the response letter and revised manuscript easier to read for the editor and reviewers, the changes are marked in "red color" for reviewers' comments. Finally, we change the order of authors.

### Data Availability Statement:

All relevant data in this study are available at https://doi.org/10.5064/F6ULCCQA

### Revisions

Thanks to the reviewers for the questions raised during the review process, we have made the following modifications in response to the additional requirements for revision comments.

1. The style of the manuscript was revised, as well as the naming of the documents and other issues.

2. Reviewed the citation format of references and revised the format of some of the literature; in addition, old literature was replaced with new ones and relevant literature was added.

3. Added a section "Acknowledgments" to the manuscript, in which the source of funding for the study is described.

4. Regarding the issue of "Competing Interests", there are no competing interests among the authors in this study.

5. Submit the corresponding minimal data set of the results described in the manuscript.

6. In the manuscript, a detailed description of the review comments and the corresponding changes were added, as described in "Responses to Reviewer".

### Responses to Reviewer

***Q1:****.* *Please ensure that your manuscript meets PLOS ONE's style requirements, including those for file naming. The PLOS ONE style templates can be found at <https://journals.plos.org/plosone/s/file?id=wjVg/PLOSOne_formatting_sample_main_body.pdf> and <https://journals.plos.org/plosone/s/file?id=ba62/PLOSOne_formatting_sample_title_authors_affiliations.pdf>.*

**Response to *Q1*:** Thanks for the editor's comments. According to the style requirements of PLOS ONE, firstly, we modified the font of the abstract and adjusted the title number; then, we added Contributorship to reflect the author's contribution in the work; finally, we adjusted the positional relationship between the figure and its citations .

***Q2:*** *Please review your reference list to ensure that it is complete and correct. If you have cited papers that have been retracted, please include the rationale for doing so in the manuscript text, or remove these references and replace them with relevant current references. Any changes to the reference list should be mentioned in the rebuttal letter that accompanies your revised manuscript. If you need to cite a retracted article, indicate the article’s retracted status in the References list and also include a citation and full reference for the retraction notice.*

**Response to Q2:** Thanks to the editor for pointing out the problem. We have made the corresponding changes. We reviewed the citation format of the references and revised the format of some documents; in addition, the old documents were replaced with new ones, and related documents were added. The effect of reference modification is as follows. ([3] [4] [6] [7] [9] [10] [14] page 22 and [23] [24] [27] [29] [30] of page 23)

*[3] Xu Z, Luo M, Kumar N, et al. Privacy-Protection Scheme Based on Sanitizable Signature for Smart Mobile Medical Scenarios[J]. Wireless Communications and Mobile Computing,* ***2020****, 2020(1):1-10.*

*[4] Kong W, Shen J, Vijayakumar P, et al. A Practical Group Blind Signature Scheme for Privacy Protection in Smart Grid[J]. Journal of Parallel and Distributed Computing,* ***2020****, 136: 29-39.*

*[6] Xu Z, He D, Vijayakumar P, et al. Efficient NTRU Lattice-Based Certificateless Signature Scheme for Medical Cyber-Physical Systems[J]. Journal of Medical Systems,* ***2020****, 44(5): 1-8.*

*[7] Ali Z, Alzahrani B A, Barnawi A, et al. TC-PSLAP: Temporal Credential-Based Provably Secure and Lightweight Authentication Protocol for IoT-Enabled Drone Environments[J]. Security and Communication Networks,* ***2021****, 2021.*

*[9] Hong H, Sun Z, Liu X. A key-insulated CP-ABE with Key Exposure Accountability for Secure Data Sharing in the Cloud[J]. KSII Transactions on Internet and Information Systems,* ***2016****,10(5):2394−2406.*

*[10] Chen L Q, Zhu Z, Wang M Y. A Threshold Group Signature Scheme for Mobile Internet Application[J]. Chinese Journal of Computers,* ***2018****, 41(5): 1052-1067.*

*[14] Lindell Y. Fast Secure Two-Party ECDSA Signing[J]. Journal of Cryptology,* ***2021****, 34(4): 1-38.*

*[23] Wang Z H, Zhang Z F. Overview on Public Key Cryptographic Algorithm SM2 Based on Elliptic Curves [J]. Journal of Information Security Research,* ***2016****, 2(11): 972-982.*

*[24] Liu X D, Zhang W F, Wang X M. Multi-Authority Attribute-based Alterable Threshold Ring Signature without Central Authority[J]. Journal of Software,* ***2018****, 29(11): 3528-3543.*

*[27] Lindell Y, Nof A. Fast Secure Multiparty ECDSA with Practical Distributed Key Generation and Applications to Cryptocurrency Custody[C]//Proceedings of the 2018 ACM SIGSAC Conference on Computer and Communications Security.* ***2018****: 1837-1854.*

*[29] Peng C, Chen J, Obaidat M S, et al. Efficient and Provably Secure Multireceiver Signcryption Scheme for Multicast Communication in Edge Computing[J]. IEEE Internet of Things Journal,* ***2019****, 7(7): 6056-6068.*

*[30] Shen J X. Certificate-based Multisignature and Its Applications[D]. Nanjing Normal University,* ***2019****.*

***Q3:*** *Thank you for stating the following financial disclosure: "Unfunded studies".*

*At this time, please address the following queries:*

*a) Please clarify the sources of funding (financial or material support) for your study. List the grants or organizations that supported your study, including funding received from your institution.*

*b) State what role the funders took in the study. If the funders had no role in your study, please state: “The funders had no role in study design, data collection and analysis, decision to publish, or preparation of the manuscript.”*

*c) If any authors received a salary from any of your funders, please state which authors and which funders.*

*d) If you did not receive any funding for this study, please state: “The authors received no specific funding for this work.”*

*Please include your amended statements within your cover letter; we will change the online submission form on your behalf.*

**Response to** ***Q3***: Thank you editor for your question. In response to the editor's questions, we have added an "Acknowledgments" section to the manuscript, and this section addresses the issue of funding sources and the role of funders in the research. The additions to the manuscript are as follows. (lines 36-41 of page 21)

***Acknowledgments***

*This work was supported by the National Natural Science Foundation of China 61373162, the Sichuan Provincial Science and Technology Support Project 2019YFG0183, and the Sichuan Provincial Key Laboratory Project KJ201402.* *The funder had no role in study design, data collection and analysis, decision to publish, or preparation of the manuscript*

***Q4:*** *Thank you for stating the following in your* ***Competing Interests*** *section: "NO authors have competing interests"。*

*Please complete your Competing Interests on the online submission form to state any Competing Interests. If you have no competing interests, please state "The authors have declared that no competing interests exist.", as detailed online in our guide for authors at http://journals.plos.org/plosone/s/submit-now*

*This information should be included in your cover letter; we will change the online submission form on your behalf.*

**Response to** ***Q4***: Thanks to the editor for the suggestion. "None of the authors had competing interests" in our study.

***Q5:*** *In your Data Availability statement, you have not specified where the minimal data set underlying the results described in your manuscript can be found. PLOS defines a study's minimal data set as the underlying data used to reach the conclusions drawn in the manuscript and any additional data required to replicate the reported study findings in their entirety. All PLOS journals require that the minimal data set be made fully available. For more information about our data policy, please see http://journals.plos.org/plosone/s/data-availability.*

*Upon re-submitting your revised manuscript, please upload your study’s minimal underlying data set as either Supporting Information files or to a stable, public repository and include the relevant URLs, DOIs, or accession numbers within your revised cover letter. For a list of acceptable repositories, please see http://journals.plos.org/plosone/s/data-availability#loc-recommended-repositories. Any potentially identifying patient information must be fully anonymized.*

**Response to** ***Q5***: Thanks to the editor for the suggestion. We have submitted a dataset of research content to https://doi.org/10.5064/F6ULCCQA.

***RQ1:*** *1. Only 10% of reference in this manuscript is recent work others are too old. Authors has to increase recent references in to 20%. Align the paper to journal format. No keyword or indexed terms in your research work.*

**Response to** ***RQ1:*** Thank you for the reviewer’s comment. To address the issue of references, we have now updated the references and increased the percentage of recent literature to 20%. In addition, we have adjusted the formatting of the paper to conform to the journal as much as possible, including adjusting formatting issues and adding some chapters. Finally, for the "Keywords" issue, although there is no "Keywords" section in LaTeX templates, published manuscripts (doi: 10.1371/journal.pone.0253454), we will add the corresponding "Keywords". The "Keywords" is given below. (lines 34-35 of page 1)

***Keywords:*** *Mobile Internet; SM2 Co-signature; Privacy Protection; Key Protection; Multi-party Computation.*

*2. This article proposed cryptography mechanism for multiple user signing. This will ensure the product verification in many applications. Kindly answer the below questions.*

***RQ2-1:****1. The scheme proposed here matches with blockchain technology, except mutiuser sign. But, aim of the work can be accomplished by blockchain permissionless (private) scheme, in which only the authenticated users can verify. Pls clarify.*

**Response to** ***RQ2-1:*** Thanks to the reviewers for their questions. We have researched the information and carefully thought about the private chain issue. We believe that the co-signature work in this paper cannot be realized by blockchain private chains. The reasons are as follows: First, the private chain is a blockchain used to control and process transactions between individuals or institutions, which is suitable for financial scenarios such as data management and auditing; while multiparty co-signing is a solution to the trust problem in data transmission, and this solution guarantees that no party can construct a complete signature private key alone, which is a guarantee for the trustworthiness of transmitted data. Secondly, in blockchain private chain, the identity of private chain nodes can be forged by evil nodes, while in multi-party co-signing, the identity is confirmed when the group public key is generated, and each co-signing party keeps its own private key separately, even if the perpetrator just constructs the same public key as the cooperating party, in the end, it will not pass the signature result verification, which is guaranteed by the properties of the elliptic curve.

***RQ2-2:*** *2. The performance analysis indicate only the runing time of the SM2 scheme. There are many security metrics available.*

**Response to** ***RQ2-2:*** Thanks to the reviewers for their suggestions. We have added a new section on security metrics " anti-forgery attack " to further illustrate the resistance of the scheme in this paper. The details of the new section are as follows. (lines 10-50 of page 17 and lines 1-8 of page 18)

***7.2.1 Anti-forgery attacks***

*Multi-party co-signing is inherently resistant to forgery attacks, During the signature process, the complete private key does not appear in the memory of any party, eliminating the risk of forgery of digital signatures due to private key leakage, and the complete signature cannot be generated without the participation of any party after the public key of the co-signing group is determined. In addition, the identity of each user is legitimate at the time of signature group public key generation, even if the forger forges the signature by impersonating a node in the signature process, the forged signature cannot pass the verification in the final signature verification process with the participation of the group public key, so the forger fails to forge the signature. Taking 3-party collaboration as an example, assume that the authenticated collaborative participants are userA{d_A_, P_A_}, userB{d_B_, P_B_}, userC{d_C_, P_C_}, and the generated legitimate group public key isP = d_A_^-1^d_B_^-1^d_C_^-1^[*]G - G. The counterfeiter forgerA attacks the participant userA, and forges information such as the same identity ID_A_, the same public key P_A_ (the private key is different d_X_) and other information. The steps for forging the signature are as follows:*

***(1) Forger forgerA requests participant chain to calculate the second part of the signature intermediate value (Sign_D)***

*① ForgerA initiates a signature, first selects the private value D0=b, calculates D1=dx*D0, and then sends D1 to the participant userB;*

*② The participant userB uses the private key dB to calculate D2=dB*D1, and then sends D2 to the participant userC;*

*③ The participant userC uses the private key dC to calculate D3=dC*D2, where D = D3 = dC* dB* dX* b, and finally returns D to the signature initiator forgerA.*

***(2) The forger forgerA calculates the first part of the signature r (Sign_r)***

*① ForgerA calculates ZA=Hv(ENTL||IDA||a||b||P||pk), and calculates M’=ZA||M;*

*② The counterfeiter forgerA calculates e = Hv(M'), and converts the text e to an integer, where Hv() is a one-way function;*

*③ The forger forgerA calculates the value K = (KX + KA + KB) mod n based on the data Ki sent by each participant, where KX = kX/dX, and then calculates the public point Q = K[*]G=(x1,y1 ), and convert the data type of x1 to integer.*

*④ Calculate the signature r=(e+x1) mod n, and return ③ if r=0 or r+k=n.*

***(3) Forger forgerA calculates the second part of signature s (Sign_s)***

*⑤ ForgerA uses the signature intermediate value D, private value b, self-calculated K value and the first part of the signature r value to calculate the second part of the signature s value, where s = (K+r)*D/b - r = (K+r)*dCdBdX – r.*

***(4) Verification of the forged signature result (r', s')***

*Verify the forged digital signature by deriving the verification formula (x1', y1') = [s']G + [t]P, where P = dA-1dB-1dC-1[*]G - G, t = (r' + s ') mod n. The detailed verification steps of the signature are described in Section 4.4.*

*Q’ = (x_1_’, y_1_’) = s’[*]G+t[*]P=s’[*]G+(r’+s’)[*]P*

*= s’[*]G+(r’+s’) (d_A_^-1^d_B_^-1^d_C_^-1^ [*]G - G)= (r’+s’) d_A_^-1^d_B_^-1^d_C_^-1^ [*]G - r’[*]G*

*= (r’ + (K+r’)*d_C_d_B_d_X_ – r’) d_A_^-1^d_B_^-1^d_C_^-1^ [*]G - r’[*]G*

*= (K* d_X_/d_A_ + r’ *d_X_/d_A_) [*]G - r’[*]G*

*= (K* d_X_/d_A_) [*]G + (r’ *d_X_/d_A_ - r’) [*]G	（11）*

*It can be seen from the verification result of the forged signature that Q’≠Q, x1≠x1’, then R≠r’, that is, the verification fails and the forged signature fails.*

***RQ3:*** *3. This work proposes a multi-party collaborative signing technique based on the SM2 digital signature algorithm from the GM/T003-2012 standard "SM2 Elliptic Curve Public Key Cryptography." Authors should, however, handle the following concerns.*

*• In this survey, the authors missed the case study. I suggested authors to add case study about signature scheme so that the international readers could follow the work and understand easily.*

*(1) Privacy-Protection Scheme Based on Sanitizable Signature for Smart Mobile Medical Scenarios*

*(2) Efficient NTRU lattice-based certificateless signature scheme for medical cyber-physical systems.*

*(3) A practical group blind signature scheme for privacy protection in smart grid*

*(4) Efficient and provably secure multireceiver signcryption scheme for multicast communication in edge computing*

*(5) TC-PSLAP: Temporal Credential-Based Provably Secure and Lightweight Authentication Protocol for IoT-Enabled Drone Environments*

*• I suggested authors should check the typo errors.*

*After answering above queries, the review paper may be considered for publication. I can recommend for Minor revision.*

**Response to** ***RQ3:*** We have followed the reviewer's suggestion and added relevant case studies of the signature scheme so that international readers can follow the work and understand easily. Among them, including the addition of application scenarios in Section 3, and the corresponding literature citations in the introduction and application scenarios. (lines 46-48 of page 4 and lines 1-19 of page 5)

*Based on the co-signature described in the introduction, we construct the following application scenarios. Taking a customer's loan application as an example, a bank has three-level administrative departments A, B, and C. When a customer makes a large loan in the bank, the approval process will be relatively strict, and two or more departments are often required to review the transaction documents level by level^[29]^, and the specific steps for multi-level signing of transaction documents by multiple departments are as follows.*

*1) The customer initiates an application for a large loan with the bank.*

*2) First, the bank receives the application, and the third-level department C reviews the customer's personal credit and other information, and if the basic information is legal, the transaction document will be signed for the first time.*

*3) Then, after receiving the transaction documents signed by the tertiary department, the secondary department B will conduct a valuation review on the client's asset information, etc. If the review is approved, the transaction documents will be signed for the second time.*

*4) Finally, the Level 1 department summarizes and finally reviews all the information required by the customer, and if there is no problem, the transaction document will be signed for the third time.*

*In the above application, which involves joint cooperation among 3 departments, more review steps and corresponding signatures may be required in practical application scenarios. Therefore, this section will detail the security model on which the digital signature is based and the ideal design goals of the scheme in this paper. The main goal of the digital signature algorithm security model is to ensure that the attacker cannot obtain the user's private data even through multiple signature interactions.*

*[3] Xu Z, Luo M, Kumar N, et al. Privacy-Protection Scheme Based on Sanitizable Signature for Smart Mobile Medical Scenarios[J]. Wireless Communications and Mobile Computing,* ***2020****, 2020(1):1-10.*

*[4] Kong W, Shen J, Vijayakumar P, et al. A Practical Group Blind Signature Scheme for Privacy Protection in Smart Grid[J]. Journal of Parallel and Distributed Computing,* ***2020****, 136: 29-39.*

*[6] Xu Z, He D, Vijayakumar P, et al. Efficient NTRU Lattice-Based Certificateless Signature Scheme for Medical Cyber-Physical Systems[J]. Journal of Medical Systems,* ***2020****, 44(5): 1-8.*

*[7] Ali Z, Alzahrani B A, Barnawi A, et al. TC-PSLAP: Temporal Credential-Based Provably Secure and Lightweight Authentication Protocol for IoT-Enabled Drone Environments[J]. Security and Communication Networks,* ***2021****, 2021.*

*[29] Peng C, Chen J, Obaidat M S, et al. Efficient and Provably Secure Multireceiver Signcryption Scheme for Multicast Communication in Edge Computing[J]. IEEE Internet of Things Journal,* ***2019****, 7(7): 6056-6068.*
